# Genetic and Global Epigenetic Modification, Which Determines the Phenotype of Transgenic Rice?

**DOI:** 10.3390/ijms21051819

**Published:** 2020-03-06

**Authors:** Xiaoru Fan, Jingguang Chen, Yufeng Wu, CheeHow Teo, Guohua Xu, Xiaorong Fan

**Affiliations:** 1 State Key Laboratory of Crop Genetics and Germplasm Enhancement, MOA Key Laboratory of Plant Nutrition and Fertilization in Low-Middle Reaches of the Yangtze River, Nanjing Agricultural University, Nanjing 210095, China; 2017203043@njau.edu.cn (X.F.); chenjingguang@caas.cn (J.C.); ghxu@njau.edu.cn (G.X.); 2 CAAS-IRRI Joint Laboratory for Genomics-Assisted Germplasm Enhancement, Agricultural Genomics Institute in Shenzhen, Chinese Academy of Agricultural Sciences, Shenzhen 518116, China; 3 Bioinformatics Center, Nanjing Agricultural University, Nanjing 210095, China; yfwu@njau.edu.cn; 4 Centre of Research in Biotechnology for Agriculture (CEBAR), University of Malaya, 50603 Kuala Lumpur, Malaysia; cheehow.teo@um.edu.my

**Keywords:** genetic, epigenetic, global methylation, transgenic, phenotype, OsNAR2.1

## Abstract

Transgenic technologies have been applied to a wide range of biological research. However, information on the potential epigenetic effects of transgenic technology is still lacking. Here, we show that the transgenic process can simultaneously induce both genetic and epigenetic changes in rice. We analyzed genetic, epigenetic, and phenotypic changes in plants subjected to tissue culture regeneration, using transgenic lines expressing the same coding sequence from two different promoters in transgenic lines of two rice cultivars: Wuyunjing7 (WYJ7) and Nipponbare (NP). We determined the expression of *OsNAR2.1* in two overexpression lines generated from the two cultivars, and in the RNA interference (RNAi) *OsNAR2.1* line in NP. DNA methylation analyses were performed on wild-type cultivars (WYJ7 and NP), regenerated lines (CK, T0 plants), segregation-derived wild-type from *pOsNAR2.1-OsNAR2.1* (SDWT), *pOsNAR2.1-OsNAR2.1*, *pUbi-OsNAR2.1*, and RNAi lines. Interestingly, we observed global methylation decreased in the T_0_ regenerated line of WYJ7 (CK-WJY7) and *pOsNAR2.1-OsNAR2.1* lines but increased in *pUbi-OsNAR2.1* and RNAi lines of NP. Furthermore, the methylation pattern in SDWT returned to the WYJ7 level after four generations. Phenotypic changes were detected in all the generated lines except for SDWT. Global methylation was found to decrease by 13% in *pOsNAR2.1-OsNAR2.1* with an increase in plant height of 4.69% compared with WYJ7, and increased by 18% in *pUbi*-*OsNAR2.1* with an increase of 17.36% in plant height compared with NP. This suggests an absence of a necessary link between global methylation and the phenotype of transgenic plants with *OsNAR2.1* gene over-expression. However, epigenetic changes can influence phenotype during tissue culture, as seen in the massive methylation in CK-WYJ7, T0 regenerated lines, resulting in decreased plant height compared with the wild-type, in the absence of a transformed gene. We conclude that in the transgenic lines the phenotype is mainly determined by the nature and function of the transgene after four generations of transformation, while the global epigenetic modification is dependent on the genetic background. Our research suggests an innovative insight in explaining the reason behind the occurrence of transgenic plants with random and undesirable phenotypes.

## 1. Introduction

Transgenic technologies allow gene transfer to completely unrelated organisms and their application in agriculture has increased the global transgenic crop cultivation to 181 million hectares [1]. In addition, as the basis of transgenic technology, tissue culture is also used for the clonal propagation and regeneration of many plants [2,3]. Although cultured material is not expected to show many genetic changes compared with the original material, there are clear examples of tissue culture material showing heritable phenotypic differences [4,5,6]. Phenotypic changes in tissue culture-derived material have been found to be caused by epigenetic changes [6,7,8]. For instance, Rhee et al. [7] demonstrated the silencing of epialleles by epigenetic modifications and showed that the pericarp color1 (*p1)* epialleles were capable of functioning in the presence of the correct trans-acting factors in maize. Furthermore, there are many reports on epigenetic changes caused by plant regeneration in rice [9], garlic [10], triticale [11], pineapple [12], torenia, and rye [13,14].

Variation in plant phenotype is determined by both genetic and epigenetic factors [4]. Epigenetics refers to the study of heritable phenotype changes without genetic alteration [15]. DNA methylation is an epigenetic mechanism that involves the transfer of a methyl group to the C5 position of cytosine and contributes to the epigenetic regulation of nuclear gene expression and to genome stability [16]. In plants, DNA methylation occurs in three sequence contexts: CG, CHG, and CHH (H=A, C or T) [16,17]. The modulation of DNA methylation in culture is crucial to regeneration outcomes: successful regenerants of *Oryza sativa* ssp. *japonica* had lower CG methylation levels than failed regenerants [9]. The regeneration process, with or without genetic transformation, affects gene regulation at the transcriptional and post-transcriptional levels and correlates with changes in DNA methylation patterns [18]. Transgenic approaches have been successfully used to produce herbicide and pest-resistant varieties in several crop species. However, there is only sporadic research concerning global DNA methylation changes in transgenic plants. Potential global DNA methylation modification occurring in transgenic plants is still largely unexplored [18,19,20,21,22,23]. There have been reports of transgenic plants from different genetic backgrounds having random and undesirable phenotypes [24,25,26]. Transgenic maize with overexpressed *DREB3,* a dehydration-responsive element-binding transcription factor, showed higher yields in some genetic backgrounds but not others [24]. Researchers have suggested that insert transgene position, metabolic imbalances and environmental constraints [24,27] could be the reasons behind undesirable phenotypes of transgenic plants. However, we suspect that epigenetic changes in transgenic lines may be another reason for the occurrence. 

Rice (*Oryza sativa* L.) is a major staple food for a large part of the global population. To determine whether genetic and global epigenetic modification influences the phenotype of transgenic rice, we used whole-genome bisulfite sequencing (WGBS) and methylated DNA immunoprecipitation sequencing (MeDIP-seq) methods to determine DNA methylation at the genomic level in various transgenic plants and their controls. The transgenic process includes tissue induction, selection pressure and insertion of the transgene [28]. We used wild-type Wuyunjing7 (WYJ7), its regenerated line (CK), and segregation-derived wild-type from *pOsNAR2.1-OsNAR2.1* (SDWT) to represent different transgenic processing, and wild-type of Nipponbare (NP), its *OsNAR2.1* overexpression line under the ubiquitin promoter (*pUbi-OsNAR2.1*), and the *OsNAR2.1* RNA interference (RNAi) NP line to represent different backgrounds and different transgenic expression. Previous studies have shown that OsNAR2.1 is a partner protein of rice high-affinity nitrate transporters (OsNRT2s) [29,30,31] and plays a key role in enabling the plant to cope with variable environmental nitrate supplies [29,30,31]; overexpression of *OsNAR2.1* can lead to an increase in grain yield and higher nitrogen-use efficiency (NUE) in rice cultivation systems [32,33]. We used *OsNAR2.1* transgenic lines as representative lines for the transgenic process.

## 2. Results

### 2.1. Transgenic Process Induces Significant Epigenetic Changes in Rice

To investigate global methylation caused by the transgenic process in plants, we used WGBS to sequence samples of four types of rice: Wuyunjing7 wild type (WYJ7), T_0_ plants regenerated from callus (CK), wild type plant derived from segregation of *pOsNAR2.1*-*OsNAR2.1* (SDWT) and *pOsNAR2.1*-*OsNAR2.1* plants. T_4_ generation SDWT plants served as true experimental controls for the *pOsNAR2.1*-*OsNAR2.1* line to study the transgene insertional effect on the epigenome as it was derived from the T_0_ *pOsNAR2.1*-*OsNAR2.1* heterozygote line (for the selection process see Supplementary Table S1). T_4_ generation *pOsNAR2.1*-*OsNAR2.1* plants with high yield and high NUE phenotypes have been described in Chen *et al*. [33]. We also used MeDIP sequencing on four more samples, including the wild type of Nipponbare (NP), the knockdown plant of *OsNAR2.1* by RNA interference (RNAi) (describe as r1 in Yan *et al*. [29]), and overexpression plant of *OsNAR2.1* by the ubiquitin promoter (*pUbi-OsNAR2.1*). The relationships between different samples are described in Table 1 as: CK = WYJ7 + callus inducing media(CIM) + shoot inducing media(SIM); SDWT = WYJ7 + CIM + SIM + selection pressure (Hygromycin, Hyg) + transformation with the *pOsNAR2.1-OsNAR2.1* construct + segregation of *pOsNAR2.1-OsNAR2.1* heterozygote line; *pOsNAR2.1-OsNAR2.1* = WYJ7 + CIM + SIM + selection pressure (Hyg) + transformation with the *pOsNAR2.1-OsNAR2.1* construct + *pOsNAR2.1-OsNAR2.1* insertion; *pUbi-OsNAR2.1* = NP + CIM + SIM + selection pressure (Hyg) + transformation with the *pUbi-OsNAR2.1* construct + *pUbi-OsNAR2.1* insertion; RNAi = NP + CIM + SIM + selection pressure (Hyg) + RNA interference (RNAi) construct. Table 1 also shows the values for raw reads, uniquely mapped reads, and normalized cytosine methylation (mC) number in each replicate of four samples for WGBS and filtered reads, aligned reads, and peaks counts of three samples for MeDIP. We sequenced the flanking DNA of *pOsNAR2.1*-*OsNAR2.1* insertion site of in *pOsNAR2.1*-*OsNAR2.1* plants and found that the insertion of the *pOsNAR2.1*-*OsNAR2.1* transgene was between LOC_Os02g49950 gene and LOC_Os02g49960 gene in chromosome 2. We also sequenced the flanking DNA of the *pUbi*-*OsNAR2.1* insertion, which was inserted in chromosome 10, between LOC_Os10g33874 and LOC_Os10g33900 in *pUbi*-*OsNAR2.1* line (Supplementary Figure S1d,e). Neither of these sites occur in high methylation areas nor in functional genes.

**Figure 1:**
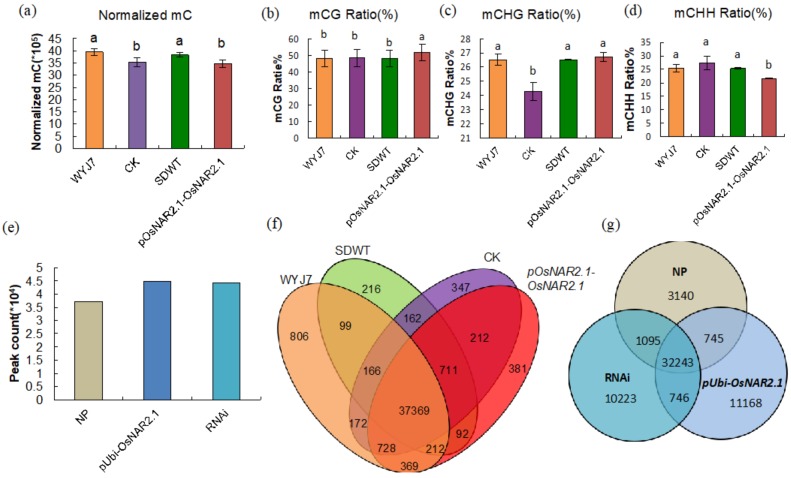
Characteristics of sequencing data (**a**) Normalized DNA cytosine methylation numbers in WYJ7, CK, SDWT and *pOsNAR2.1-OsNAR2.1* samples. (**b**-**d**) mCG, mCHG and mCHH ratio in WYJ7, CK, SDWT and *pOsNAR2.1-OsNAR2.1* samples. (**e**) Peak count of methylation in NP, *pUbi-OsNAR2.1* and RNAi. (**f**) Venn diagram of unique and shared genes in CpG methylation states for WYJ7, CK, SDWT and *pOsNAR2.1-OsNAR2.1* samples. (**g**) Venn diagram of peaks in NP, RNAi and *pUbi-OsNAR2.1* samples.

Our analysis of the sequencing data showed that the total normalized methylated cytosine (mC) numbers differed significantly among the four samples (Figure 1a), with normalized mC counts of approximately 3.9, 3.5, 3.8, and 3.5 million (M) for WYJ7, CK, SDWT, and *pOsNAR2.1-OsNAR2.1*, respectively. The result suggests that the tissue culture (CK) and the transgenic process (*pOsNAR2.1-OsNAR2.1*) of WYJ7 both lead to global DNA hypomethylation. In contrast to CK and *pOsNAR2.1-OsNAR2.1* plants, the mC level in SDWT plants returned to the WT level (WYJ7) after four selfing generations in the field. Since the CK and SDWT both went through regeneration, the result suggested the loss of the transgene in generation increases the methylation in SDWT. Simultaneously, the *pOsNAR2.1-OsNAR2.1* lines showed a significantly higher percentage of symmetric CG sites and lower percentage of asymmetric CHH sites, and CK lines showed a lower percentage of symmetric CHG sites instead (Figure 1b–d), which indicates that while both *pOsNAR2.1-OsNAR2.1* and CK lines show a decrease in global DNA methylation the sites of mC changes were different. Interestingly, in the other two independent transgenic lines, MeDIP sequencing data showed that peak counts in both *pUbi-NAR2.1* and RNAi lines increased compared with NP (Figure 1e). The results suggest that the tissue culture process leads to a decrease in global methylation, but the transgenic process leads to different global methylation changes in different rice backgrounds. 

### 2.2. Both Tissue Culture and Transgenic Processes Can Induce Unique Methylation Changes in Genic Regions

The Venn diagram of WGBS sequencing shows the unique and shared CpG methylation areas of 42,042 genes in the WYJ7, CK, SDWT and *pOsNAR2.1*-*OsNAR2.1* samples (Figure 1f). The results showed that 88.9% of genes in these four samples shared the same CpG methylation area. CK, SDWT, and *pOsNAR2.1*-*OsNAR2.1* had 347, 216, and 381 genes with unique methylation areas, respectively. If only compared with wild-type WYJ7, CK, SDWT, and *pOsNAR2.1*-*OsNAR2.1* showed 1432, 1181, and 1396 genes with unique methylation areas. The Venn diagram analysis of the methylation peaks of the MeDIP-seq data sets for NP, RNAi, and *pUbi-OsNAR2.1* samples showed that the majority of the peaks, approximately 32,000, are shared by the three samples (Figure 1g). Both RNAi and *pUbi-OsNAR2.1* samples had 10,223 and 11,168 unique peaks, respectively (Figure 1g). The results indicate that both tissue culture and the transgenic processes could induce unique methylation changes in genic regions although neither knockdown nor overexpression of *OsNAR2.1* in transgenic plants caused the majority of DNA methylation changes. 

### 2.3. Transgenic Process Leads to a Higher Number of Hyper-DMRs Than Hypo-DMRs

**Figure 2:**
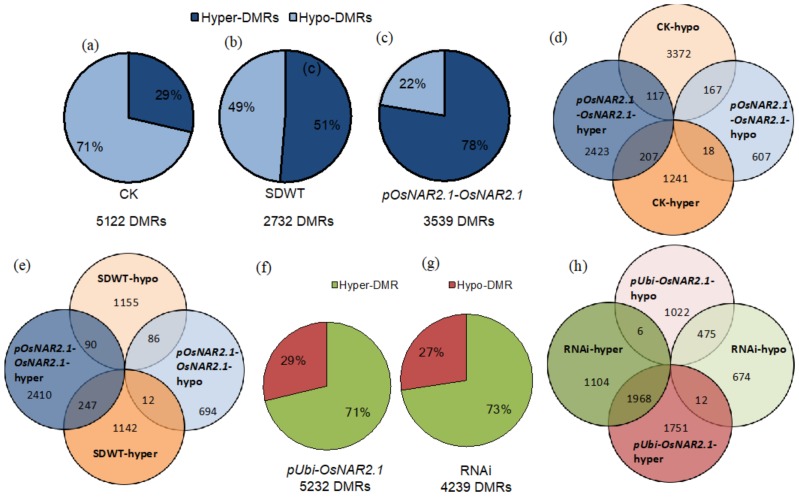
Characteristics of DMRs. (**a**-**c**) Total number of DMRs and breakdown of hyper- and hypo-DMRs in CK, SDWT and *pOsNAR2.1-OsNAR2.1* samples. (**d**) Venn diagram of unique and shared hyper- and hypo-DMRs in *pOsNAR2.1-OsNAR2.1* and CK samples. (**e**) Venn diagram of unique and shared hyper- and hypo-DMRs in *pOsNAR2.1-OsNAR2.1* and SDWT samples. (**f**,**g**) Total number of DMRs and breakdown of hyper- and hypo-DMRs in *pUbi-OsNAR2.1* and RNAi samples. (**h**) Venn diagram of unique and shared hyper- and hypo-DMRs in *pUbi-OsNAR2.1* and RNAi samples.

We analyzed the differentially methylated regions (DMRs) of wild-type and transgenic lines and found that there were more hypo-DMRs (71%) in CK lines but more hyper-DMRs (78%) in transgenic lines in *pOsNAR2.1-OsNAR2.*1 under WYJ7. Similar results were observed in *pUbi-OsNAR2.1* and RNAi under NP (71% and 73%) and, for the SDWT line, the percentage of the hypo- and hyper- DMRs were very close (49% and 51%) (Figure 2a–c,f,g). In spite of global DNA methylation caused by the transgenic process being background sensitive, the insertion of the transgene was able to induce more hyper-DMRs than hypo-DMRs in both backgrounds. CK and the three transgenic lines contained 5122, 3539, 5253, and 4239 DMRs, respectively, which were considerably more comparable with the 2732 DMRs of SDWT. There were few common DMRs between these hypo- and hyper-DMRs in CK and *pOsNAR2.1-OsNAR2.1* lines, nor in SDWT and *pOsNAR2.1-OsNAR2.1* lines (Figure 2d,e). However, there were nearly 2000 common hyper-DMRs and nearly 500 common hypo-DMR between the *pUbi-OsNAR2.1* and RNAi lines (Figure 2h), caused by insertion of the transgene, rather than by the expression of *OsNAR2.1*. 

### 2.4. Tissue Culture Process Causing Random Epigenetic Changes

**Figure 3:**
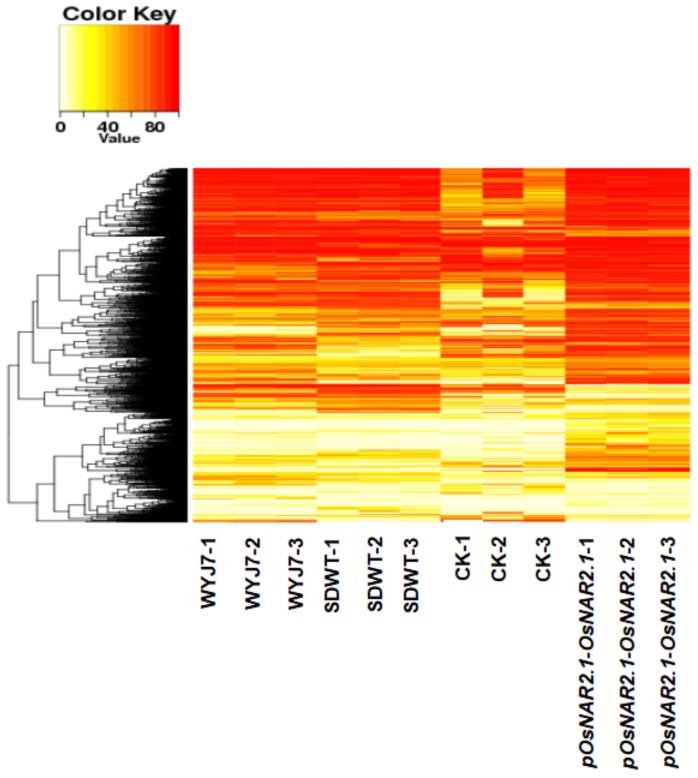
Heatmap for DMRs methylation level cluster. Heatmap representation of hierarchical clustering based on CG methylation levels within DMRs. Rows represent all DMRs identified and columns represent the samples.

We analyzed the differentially CpG methylated regions of five comparison groups: WYJ7 and SDWT, WYJ7 and CK, WYJ7 and *pOsNAR2.1-OsNAR2.1*, SDWT and *pOsNAR2.1-OsNAR2.1*, and CK and *pOsNAR2.1-OsNAR2.*1. We found that the mutual DMRs differed among them and calculated the CpG methylation level in these DMRs in different replicates of different samples. The clustered heatmap is shown in Figure 3. The heatmap shows that the DMR clustering pattern is similar between wild-type and lines subjected to transgenic manipulation (SDWT and *pOsNAR2.1-OsNAR2.1*), whereas a more variable pattern is observed for CK replicates. The result shows that different CpG clusters of common DMRs were detected in different CK lines regenerated from the same tissue culture process with the same explant WYJ7 seeds (Figure 3). This suggests that the tissue culture process could randomly alter the epigenetic status. On the other hand, a tissue culture process followed by a transgenic process tends to lead to a more consistent CpG methylation level clustering pattern (Figure 3), suggesting that the influence of the transgenic process on the rice epigenome is stronger than the influence of the tissue culture process.

### 2.5. Both Tissue Culture and Transgenic Process Can Change the Phenotype of Plants

**Figure 4:**
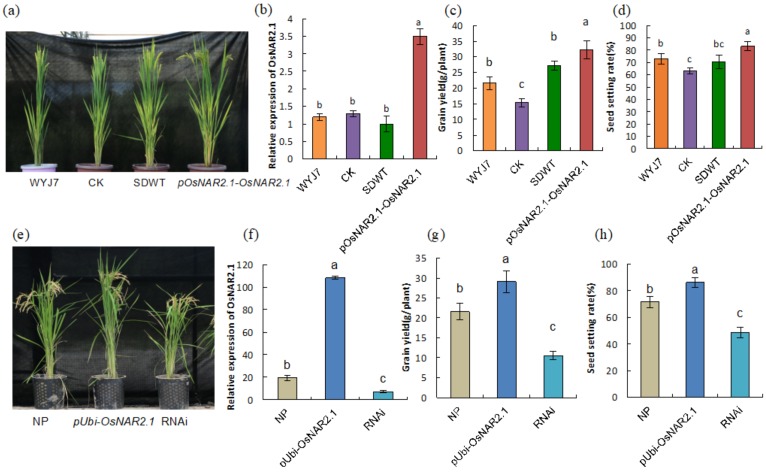
Characteristics of phenotype (**a**) Gross morphology of WYJ7, CK, SDWT and *pOsNAR2.1-OsNAR2.1.* (**b**) Real-time quantitative RT-PCR analysis of *OsNAR2.1* expression in WYJ7, CK, SDWT and *pOsNAR2.1-OsNAR2.1* lines. Error bars: SD (*n* = 3 plants). (**c**) Grain yield for WYJ7, CK, SDWT and *pOsNAR2.1-OsNAR2.1* plants grown in the field. Error bars: SD (*n* = 5 plants). Significant differences between WYJ7 and transgenic lines are indicated by different letters (P < 0.05, one-way ANOVA). (**d**) Seed setting rate for WYJ7, CK, SDWT and *pOsNAR2.1-OsNAR2.1* plants grown in the field. Error bars: SD (*n* = 5 plants). Significant differences between WYJ7 and transgenic lines are indicated by different letters (*p* < 0.05, one-way ANOVA). (**e**) Gross morphology of wild-type of NP, *pUbi-OsNAR2.1* and RNAi. (**f**) Real-time quantitative RT-PCR analysis of *OsNAR2.1* expression in NP, *pUbi-OsNAR2.1* and RNAi lines. Error bars: SD (*n* = 3 plants). (**g**) Grain yield for NP, *pUbi-OsNAR2.1* and RNAi plants grown in the field. Error bars: SD (*n* = 5 plants). Significant differences between NP and transgenic lines are indicated by different letters (*p* < 0.05, one-way ANOVA). (**h**) Seed setting rate for NP, *pUbi-OsNAR2.1* and RNAi plants grown in the field. Error bars: SD (*n* = 5 plants). Significant differences between NP and transgenic lines are indicated by different letters (*p* < 0.05, one-way ANOVA).

We planted all the wild-type and transgenic lines in the field and estimated their gene expression, grain yield, and seed setting rate (Figure 4). For the lines used in the WGBS analysis, the phenotype showed that the plant height was significantly shorter in the CK line and significantly taller in the *pOsNAR2.1-OsNAR2.1* line compared with WYJ7, whereas there was no significant change in the SDWT line (Figure 4a, Supplementary Table S2). The total tiller number was higher in *pOsNAR2.1-OsNAR2.1* line with no significant difference in the CK and SDWT lines (Supplementary Table S2). For the expression of *OsNAR2.1*, while there were no significant differences between the WYJ7, CK and SDWT lines, expression was significantly higher in the *pOsNAR2.1-OsNAR2.1* line (Figure 4b). The grain yield and seed setting rate were significantly lower in the CK line and significantly higher in the *pOsNAR2.1-OsNAR2.1* line compared with WYJ7, with no significant difference in the SDWT line (Figure 4c,d). For the lines used in MeDIP analysis, both the plant height and tiller number (Figure 4e, Supplementary Table S3) were higher in the *pUbi-OsNAR2.1* line and lower in the RNAi line compared with NP. The relative expression of *OsNAR2.1*, grain yield, and seed setting rate were also higher in the *pUbi-OsNAR2.1* line and lower in the RNAi line, compared to NP (Figure 4f–h). As a summary of the global methylation, genetic and phenotype changes status of all materials Table 2 shows that global methylation changes of transgenic lines are dependent on the genetic background. For the CK line, the trend of changes of the phenotypes is same as global methylation, and for all the transgenic lines, the trend for phenotypic change appears the same as the expression of the transgene, but the percentages of the increases in overexpression lines are quite different. We consider that methylation change is one of the reasons causing the difference. These results suggest that both regeneration and the transgenic process can change the phenotype of plants, with or without gene insertion.

## 3. Discussion

DNA methylation can provide additional heritable information beyond that of the DNA sequence in plant genomes [2]. Tissue culture is considered a stressful environment and thus trigger epigenetic changes in plants [34]. Culture-induced DNA methylation has been found in different species, including rice, maize, and barley [35,36,37]. Furthermore, many studies have shown that regeneration under various selection stresses or from various donor tissues induced changes in methylation patterns [38,39,40]. When uniform callus donor tissue was used in an *Agrobacterium*-mediated transformation procedure in rice [41,42], there was no difference in methylation among donor tissues between experiments. It has previously been reported that tissue culture reduces mC in rice, and this reduction in mC is stable from T_2_ to T_6_ generation of regenerated plants [18]. Our sequence data confirmed that the tissue culture process leads to a reduction in global DNA methylation in CK and that this reduction was maintained in the *pOsNAR2.1-OsNAR2.1* line at least until the T_4_ generation (Figure 1a). Moreover, we showed that the global methylation level returned to a level similar to that of WYJ7 after removal of the transgene by a segregation process in the SDWT line. Since Stroud et al. [18] confirmed that the loss of methylation in regenerated plants is stable across generations, our results suggest that the global methylation status in the SDWT is more unstable and is unable to maintain the mC decrease across the generations. However, the insertion of the transgene stabilized the massive loss of mC in *pOsNAR2.1-OsNAR2.1*. The tissue culture process can cause the loss of methylation in both WYJ7 and NP (Figure 1a) according to both our results and those of Stroud, et al. [20], but *pOsNAR2.1-OsNAR2.1* in WYJ7 showed stabilization of the loss rather than *pUbi-OsNAR2.1* in NP. Therefore, we consider that overexpression of *OsNAR2.1* is not the reason behind the stabilization. We prefer the explanation that the unstabilized methylation of SDWT was caused by the double genetic change, gaining and losing the transgene during generation. Furthermore, the way insertion of the transgene alters global methylation appears to depend on the rice genetic background, since global methylation reduction was observed in the *pOsNAR2.1-OsNAR2.1* WYJ7 line whereas it was increased in the *pUbi-NAR2.1* and RNAi NP lines. 

We found global methylation decreased in the WYJ7 *OsNAR2.1-*overexpression line, while increased *OsNAR2.1*-overexpression, and RNAi lines in the NP. We suggest that rice varieties have different sensitivities to DNA methylation in the transgenic process. It has been reported that there are extensive variations in DNA methylation among plant inbred lines, and that DNA methylation can provide unique information in explaining variation of phenotype in maize [43]. Vilperte et al. [5] reported that the methylation status of genes showed significant differences in four different maize backgrounds with the same transgene. In our results, although in both the *pOsNAR2.1-OsNAR2.1* and *pUbi-OsNAR2.1* lines, plant height and yield of per plant were significantly higher than in the wild-type, but the plant height increased 4.69% and 17.36%, and yield of per plant increased 49.07% and 34.97%, respectively, in WYJ7 and NP (Table 1, Supplementary Tables S2 and S3). Even overexpression of the same gene was able to cause different phenotypes in different backgrounds. This result could be caused by several possible factors. The first of these is the original traits of the two wild types: WYJ7 is photo-sensitive late-maturing japonica rice and NP is photo-insensitive early-maturing japonica rice [44,45]. The two wild-types are, therefore, affected differently by the circadian clock. The circadian clock regulates NO_3_^-^ uptake and usage, and thus the expression of *OsNAR2.1* [30,46]. A second factor is the promoter of the transgene. It is well known that different promoters have different effects on transgenes [47]. In our research, the expression of *OsNAR2.1* increased around 2.5 times with the *OsNAR2.1* native promoter and increased four times with the *Ubi* promoters. Wang et al. [48] reported that overexpressed auxin-inducible gene (*ARGOS*) increased plant height in *Arabidopsis* under the 35*s* promoter but showed no phenotypic change under the *Ubi* promoter. The third factor is background. There have been many reports of the same transgene showing different phenotypes in plants from a variety of genetic backgrounds. It has been reported that, while the *DREB3* transgene was detected in wheats from four different genetic backgrounds, only three lines expressed the transgene, and only two showed phenotypes of higher yield [24]. Knockout of *OsNramp5*, a member of the natural resistance-associated macrophage protein (NRAMP) family, decreased yield in Xidao 1, a japonica rice cultivar, but did not alter yield in indica hybrid rice [26,49]. Even knockout of the same gene in different backgrounds with same genome also resulted in phenotypic variation, as shown by the findings of Yang et al [50]: knockout of *OsNramp5* using CRISPR/Cas9 in two japonica varieties, Nanjing 46 (NJ46) and Huaidao 5 (HD5), resulted in similar plant height, grain number, and seed setting rate, but with increased panicle number in NJ46 but not in HD5. Researchers surmised that damage to the recipient genome caused by insertion fragments, efficiency of the transgene promoter, metabolic imbalances and environmental constraints could be the reasons behind undesirable phenotypes of transgenic plants [24,27,51], while we suspected that the insertion of the transgene causing different methylation change in different backgrounds could be another reason.

The Venn diagram of genes in the methylation region in WYJ7, SDWT, CK, and *pOsNAR2.1-OsNAR2.1* showed that all three had over 1000 unique methylation genes compared with WYJ7 (Figure 1f), and all had 200–400 unique methylation genes compared to each other. The results suggest that, compared with WYJ7, all three samples (CK, SDWT, and *pOsNAR2.1-OsNAR2.1*) have abundant genes with different degrees of methylation, but not all three lines showed phenotypic changes (Figure 4a). In spite of having these differentially methylated genes, SDWT still showed a similar phenotype to WYJ7. These results suggest that the global methylation status change had a stronger influence on plants than changes in the methylation of individual genes.

Our results showed that the regenerated line CK had more hypo-DMRs than hyper-DMRs and that all three transgenic lines had more hyper- than hypo-DMRs, compared to the wild-type. However, in SDWT, the percentage of hyper- and hypo-DMRs are similar, 51% and 49%, respectively. The total number of DMRs in SDWT (2732 DMRs) was much less than that in CK (5122 DMRs) and the three transgenic lines (3539 DMRs in *pOsNAR2.1-OsNAR2.1*, 5232 DMRs in *pUbi-OsNAR2.1* and 4239 DMRs in RNAi) (Figure 2a–c,f,g). Stroud et al. [18] reported that all of their 12 regeneration lines had much more hypo-DMRs than hyper-DMRs, confirming our results. Therefore, it is possible that the reprogramming process could cause an abundance of hypo-DMRs, while insertion of the gene could cause hyper-DMRs instead, regardless of the function of the inserted gene. Furthermore, for the SDWT lines, WT went through both reprogramming and transgene processes but after segregation, it was found that this double genetic change resulted in nearly 3000 DMRs with the numbers of hyper- and hypo-DMRs returning to the baseline level of WYJ7. Since the phenotype of SDWT did not show significant differences compared to WYJ7, this indicated that the balance of hyper- and hypo-DMRs could be an important factor for the stability of the epigenetic status.

We found that the methylation status in the CK line regenerated from the tissue culture process gave rise to a more variable DMR methylation level clustering pattern than in the CK replicates. This is similar to the observations of Hsu et al. [9]. These authors reported that methylomes in different stages of callus showed a high level of variability and the global methylome demonstrated a prominent genetic/cultivar-related impact. Kaeppler et al. [4] showed that epigenetic modifications of genomic DNA are less stable in culture. Interestingly, both the transgenic line (*pOsNAR2.1-OsNAR2.1*) and the wild-type lines derived from the segregation process of *pOsNAR2.1-OsNAR2.1* (SDWT) showed a more consistent DMR methylation level clustering pattern. This suggests that the transgenic process may play a role in stabilizing the global methylation status of tissue cultured plants. 

However, it is possible that the CK line has a genetic change in the genome in addition to epigenetic changes. For example, there have been many reports of the occurrence of transposons, such as *Tos17,* in regenerants [34,52]. To clarify the effects of epigenetic changes on the CK phenotype, we have listed the methylation sequence data and agronomic traits of each CK line from three independent calli in Supplementary Table S4. We found that, despite the differences between individuals, there is an overall consistency in the data of CK replicates (Supplementary Table S4). The Table shows that normalized mC number, mCHG ratio of epigenetic and plant height, panicle length, grain number per panicle, and yield of phenotype are all significantly lower in CK. Even if we cannot exclude possible genetic change in CK, the changes, at least, did not influence the phenotypes of the individual CK replicates in our study. It has also been reported that genetic variation during tissue culture is related to the CCGG target, which suggests that genetic and epigenetic changes in regenerants are relevant [34,53,54]. These changes could be the reason for the clustering of variable DMR methylation levels in CK but not causing phenotypic changes.

Previous research has reported that phenotypes are influenced by both genetic and epigenetic mechanisms [20,55,56]. Epigenetic modifications caused by the tissue culture process occur in an apparently random manner and it is thus difficult to predict phenotypic changes resulting from these modifications [4,5,7,57,58]. Here, we summarized global methylation, genetic and phenotype changes of five plant materials from two varieties, as shown in Table 2. The results indicate that, in the transgenic lines (*pOsNAR2.1-OsNAR2.1*, *pUbi-OsNAR2.1,* and RNAi), the phenotype is mainly determined by the nature and function of the transgene, but the global methylation change in transgenic lines is determined by the rice background rather than the function of the transgene, which may cause the difference between the phenotypic changes in the two overexpression lines. However, the CK lines showed fewer phenotypic changes, with the methylation status being significantly decrease from WYJ7 as result of the tissue culture process and showing DMR variation between replicates (Figure 3). 

## 4. Materials and Methods 

### 4.1. Plant Materials for Sequencing 

For WGBS sequencing, we used the shoots of wild-type *Oryza sativa* L. ssp. *japonica* cv. Wuyujing7 (WYJ7), T_0_ plants regenerated from tissue culture (CK), T_4_-generation plants carrying an insertion of the *pOsNAR2.1-OsNAR2.1* construct (*pOsNAR2.1-OsNAR2.1*) as described as Ox1 in Chen et al. [33] (see Supplementary Figure S1 and Table S2 for the transformation construct and plant growth data), and T_4_-generation wild-type line lacking the transgene that derived from the segregation process of the T_0_ *pOsNAR2.1-OsNAR2.1* heterozygote line (SDWT, see Supplementary Tables S1 and S2). T_1_ generation of Ox1-1 (AA) and Ox1-3 (aa) lines shown in Supplementary Table S1 were renamed as *pOsNAR2.1*-*OsNAR2.1* and SDWT and their T_4_ plants were used for further WGBS experiment. 

The WYJ7, SDWT, and *pOsNAR2.1-OsNAR2.1* were sterilized for 30 min with 10% (*v*/*v*) hydrogen peroxide, washed thoroughly with deionized water and then grown in water. CK lines were moved from root medium to water after germination. All samples were collected when all four lines grew to two leaves and one heart period. DNA from each plant was sequenced with three replicates. Before sequencing, we tested for the T-DNA insertion loci of *pOsNAR2.1-OsNAR2.1* (Supplementary Figure S1d). The primers used for TAIL-PCR are listed in Supplementary Table S5.

We used MeDIP-seq to examine three types of plants: wild-type of *Oryza sativa* L. ssp. *japonica* cv. Nipponbare (NP), the T_8_-generation plant of knockdown plant of *OsNAR2.1* by RNA interference (RNAi) (describe as r1 in Yan et al. [29]), and the T_8_-generation *pUbi-OsNAR2.1* overexpression plant. The transformation constructs and plant growth data are shown in Supplementary Figure S1 and Table S3. We harvested the mixed samples of the first leaf blade, culm and panicles at the anthesis stage for MeDIP-seq (Figure 4e). Details of the growth conditions of the plants in soil are listed in Supplementary Table S3. The level of *OsNAR2.1* expression was reduced in T_8_ generation RNAi plants to one half of the WT, whereas was five times higher in the *pUbi-OsNAR2.1* T_8_ generation transgenic line (Figure 4f). Other growth characteristics of RNAi and *pUbi-OsNAR2.1* transgenic lines are described in Supplementary Table S3. We tested for T-DNA insertion in three samples (Supplementary Figure S1e); the primers for TAIL-PCR are listed in Supplementary Table S5.

### 4.2. Growth Conditions of Plants in the Field

All materials were grown in plots at the Baguazhou base of Nanjing Agricultural University in Nanjing, Jiangsu. Nanjing is located in a subtropical monsoon climate zone. The pH of the soil is 6.5, and chemical properties included 0.91 g/kg total N content; 18.91 mg/kg available phosphorus (P) content; 185.67 mg/kg exchangeable potassium (K) and 11.56 g/kg organic matter. We applied Ca(H_2_PO_4_)_2_, 30 kg P/ha and KCl, 60 kg K/ha as basal applications to the plots three days before transplanting. We use urea as N fertilizer, and applied 40% before transplanting, 30% at tilling, and 40% before the heading stage. Plots size was 2 × 2.5 m and the seedlings planted in a 10 × 10 array [32,59,60,61,62]. We randomly chose five seedlings from each plot, avoiding those on the edges. The agronomic characters of plant height, total tiller number per plant, panicle length, seed setting rate per plant, grain weight per panicle, grain number per panicle and yield per plant were measured at the maturity stage. Plant height indicated the height of the highest panicle. One panicle from each plant was chosen for calculating the panicle length, grain weight per panicle and grain number per panicle [33]. The agronomic traits of the samples are listed in Supplementary Tables S2 and S3. 

### 4.3. Gene Expression Analysis

Total RNA was extracted from leaf tissue using TRIzol reagent (Vazyme Biotech Co, Ltd., People’s Republic of China). DNase I-treated total RNAs were subjected to reverse transcription (RT) with HiScript II Q Select RT SuperMix for qPCR (+gDNA wiper) kit (Vazyme Biotech Co, Ltd., People’s Republic of China) according to the manufacturer’s instructions. Quantitative assays were performed using AceQTM qPCR SYBR Green Master Mix (Vazyme Biotech Co, Ltd., China). Relative expression level is normalized to the amount of *OsActin* (LOC_Os03g50885) in the same sample and presented as 2^–^^△CT^. All primers used for RT-qPCR are listed in Supplementary Table S6.

### 4.4. T-DNA Insertion Loci Analysis

Leaf tissues harvested from the plants at 10 days were grown in water. Genomic DNA isolation was performed using the CTAB extraction procedure [63]. T-DNA insertion loci of two overexpression transgenic lines were determined by TAIL-PCR following the procedures previously described [64]. The primers are listed in Supplementary Table S5. 

### 4.5. WGBS and Data Analyses 

Genomic DNA was extracted from 10 day-old seedlings and shipped to the Anoroad Genome Company (Beijing, China) for WGBS. WGBS library preparation and sequencing was performed by the Anoroad Genome Company (Beijing, China).

After downloading, the raw reads were filtered and trimmed to obtain clean reads and the available data were compared with the reference genome of *Oryza_sativa Japonica* IRGSP-1.0 to obtain the alignment results using Bismark (v0.9.0) [65]. The uniquely mapped reads will be used to call the methylated cytosines (mC) in highly enriched regions. For each cytosine site, the methylation level (%) was calculated by: 100 × (reads-supported methylation)/(total reads depth for the site). For the methylated region, the methylation level (%) was calculated by: 100 × methylation level of all cytosine sites in the region / the total number of cytosine sites in the region. The conversion ratio of Lambda DNA was around 99.5% to 99.6% in all samples. Valid coverage of methylated cytosine is the percentage of methylated C among all C on the reference genome, is associated with methylation level. C sites with read depths less than 5 are eliminated firstly. Then, for each C site, valid coverage is calculated by for all C or C within each pattern (CG, CHG and CHH). Coverage is the count of C sites with more than 5 depth divided by total number of C site by its pattern. Normalized mC number (Figure 1, Table 1) was calculated by: total mC number/valid coverage of methylated cytosine. Relative data are listed in Supplementary Table S7.

The differential analysis of methylation was performed at the region-base level, differentially methylated regions (DMRs). Aberrant DNA methylation was compared with the control group, an increased methylation pattern was defined as hyper-methylation, whereas a decreased methylation pattern was defined as hypo-methylation. DMR detection was performed using a methylKit and eDMR package [66] to estimate the boundary of CpG islands with a mixed bimodal normal distribution assumption of the distance between CpGs. 

### 4.6. MeDIP-Seq and Data Analysis

MeDIP-seq for this study was performed by KangChen Bio-tech, Shanghai, China. Sequencing library preparation and data analysis were as performed described by Ding et al. [67]. The available data were aligned to the reference genome of *Oryza_sativa. Japonica* IRGSP-1.0 using BOWTIE software (V2.1.0) [68]. MeDIP peaks were identified by MACS2 [69], and statistically significant MeDIP-enriched regions (peaks) were identified by comparison to a Poisson background model (cut-off q-value = 10^–2^). DMRs in MeDIP dataset was identified by diffReps [70] (*p* < 0.0001).

### 4.7. Statistical Analysis

The data of two groups were analyzed by paired sample Student’s test (*t*-test) and the data of more than two groups were analyzed by Tukey’s test of one-way analysis of variance (ANOVA). Statistically significant differences at *P* < 0.05 between samples were indicated by different letters on the histograms or after mean values. All statistical evaluations were conducted using the IBM SPSS Statistics version 23 software (SPSS Inc., Chicago, IL, USA). 

## 5. Conclusions

We found that both tissue culture and transgenic processes cause global methylation changes. The tissue culture process generally leads to a reduction in global methylation whereas the transgenic process causes global methylation changes that are dependent on the background. Transgenes could cause the global methylation decrease in WYJ7 and increase in NP. In addition, tissue culture caused abundant hypo-DMRs while the transgenic process caused hyper-DMRs. Epigenetic changes such as large amounts of methylation can influence phenotype during the tissue culture process. This happened in CK, resulting in small-sized plants compared to the wild-type WYJ7, even with no transformed gene. In the transgenic lines, while the phenotype is mainly determined by the nature and function of the transgene, but the global epigenetic modification in transgenic lines occurred and resulting in different patterns of changes in plants of different genetic backgrounds. Our results indicate a potential reason behind the occurrence of transgenic plants with random and undesirable phenotypes.

## Figures and Tables

**Table 1:** Samples description in this study.

Sample	Description	Raw Reads	Uniquely Mapping Reads	Normalized mC Count
WT-WYJ7-1	Wild type Wuyujing 7 cultivar (WYJ7)	140,469,848	114,222,086	396,005
WT-WYJ7-2		140,314,960	114,019,328	380,987
WT-WYJ7-3		137,035,182	111,328,844	406,068
CK1	T0 generation plant of WYJ7 +callus inducing media (CIM) + shoot inducing media (SIM)	115,760,650	84,978,096	340,144
CK2		115,709,516	83,108,206	344,139
CK3		135,865,342	98,592,558	375,679
SDWT1	T4 generation plant of WYJ7+CIM+SIM+ selection pressure (Hyg) + transformation of *pOsNAR2.1*-*OsNAR2.1* construct + segregation of *pOsNAR2.1-OsNAR2.1* heterozygote line	134,716,330	109,964,522	376,469
SDWT2		127,794,020	104,131,328	393,058
SDWT3		138,032,054	112,763,894	377,411
*pOsNAR2.1-OsNAR2.1-*1	T4 generation plant of WYJ7+CIM+SIM+selection pressure (Hyg) + transformation of *pOsNAR2.1*-*OsNAR2.1* construct + one more *pOsNAR2.1*-*OsNAR2.1* insertion.	144,544,940	79,464,658	354,464
*pOsNAR2.1-OsNAR2.1-*2		109,740,316	73,616,122	328,232
*pOsNAR2.1-OsNAR2.1-*3		136,405,336	100,950,580	356,844
		Pass Filtering Reads	Reads Aligned Reads	Peaks count
WT-NP	Wild type Nipponbare cultivar (NP)	13,047,179	12,798,555	37,223
*pUbi-OsNAR2.1*	T8 generation plant of NP+CIM+SIM+ selection pressure (Hyg)+transformation of *pOsNAR2.1-OsNAR2.1* vector+*pUbi*-*OsNAR2.1* insertion	20,671,931	20,360,102	44,902
RNAi	T8 generation plant of NP+CIM+SIM+ selection pressure (Hyg)+RNA interference (RNAi) constructs	18,722,855	18,428,653	44,307

**Table 2:** Characteristics of methylation and genetics in samples.

	Variety	WYJ7			NP	
		CK	SDWT	*pOsNAR2.1-OsNAR2.1*	*pUbi-OsNAR2.*1	RNAi
	Global methylation	↓	NS	↓	↑	↑
Genetic	Reprograming	+	+	+	+	+
	Transgenic	**NS**	**NS**	+	+	+
	Exogenous Gene	**NS**	**NS**	1	1	1
	Expression of transgene	**NS**	**NS**	↑	↑	**NA**
Phenotype	Plant height	↓ 6%	**NS**	↑ 5%	↑ 17%	↓ 18%
	Yield(g/plant)	↓ 28%	**NS**	↑ 49%	↑ 35%	↓ 51%
	Seed setting rate	↓ 13%	**NS**	↑ 14%	↑ 21%	↓ 32%

Note: NS means no significant difference. NA means not applicable. + means have, 1 means gene number, arrowhead indicates upregulate and downregulate, red indicates upregulate and green indicates downregulate.

## Data Availability

The WGBS and MeDIP dataset created for this publication are available in the NCBI GEO repository with accession number GSE94319.

## Supplementary Materials

Supplementary materials can be found at https://www.mdpi.com/1422-0067/21/5/1819/s1.
