# Supplementary material for: Genetic and Global Epigenetic Modification, Which Determines the Phenotype of Transgenic Rice?
Source: Int J Mol Sci. 2020 Mar 6;21(5):1819. doi: 10.3390/ijms21051819 (PMC7084647; doi:10.3390/ijms21051819)
Supplement: Supplementary file 1 [file ijms-21-01819-s001.pdf]

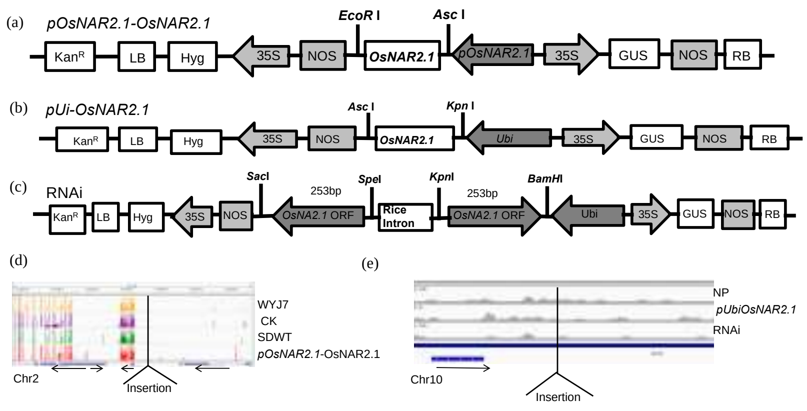

Figure S1. (a)Diagram of *pOsNAR2.1-OsNAR2.1* constructs. LB, left border; RB, right border; 35S, cauliflower mosaic virus 35S promoter; pOsNAR2.1, *OsNAR2.1* promoter; NOS, nopaline synthase terminator. (b)Diagram of RNA interference (RNAi) constructs. LB, left border; RB, right border; 35S, cauliflower mosaic virus 35S promoter; OsNAR2.1 ORF: Open Reading Frame of OsNAR2.1 gene; Ubi, Ubiquitin promoter; NOS, nopaline synthase terminator. (c)Diagram of *pUbi-OsNAR2.1* constructs. LB, left border; RB, right border; 35S, cauliflower mosaic virus 35S promoter; pOsNAR2.1, *OsNAR2.1* promoter; NOS, nopaline synthase terminator. (d) Position of *pOsNAR2.1-OsNAR2.1* insertion in the genome and the methylation level at the upstream and downstream of transgene position in WYJ7, CK, SDWT and pOsNAR2.1-OsNAR2.1 plants. (e) Position of pUbi-OsNAR2.1 insertion in the genome and the methylation level at the upstream and downstream of transgene position in NP, pUbi-OsNAR2.1 and RNAi plants.

Table S1. Copy numbers of *pOsNAR2.1-OsNAR2.1* over-expressing T0 and T1 plants in the WYJ7 background.

pOsNAR2.1-OsNAR2.1 T0 lines	Relative quantity	Absolute quantity	Copy numbers	Genotype
WYJ7		0.04	0	aa
OE1	0.84	0.62	1	Aa
OE2	1.02	0.78	1	Aa
OE3	0.24	0.82	1	Aa
pOsNAR2.1-OsNAR2.1 T1 lines	Relative quantity	Absolute quantity	Copy numbers	Genotype
OE1-1	2.12	1.64	1	AA
OE1-2	2.05	1.84	1	AA
OE1-3	0.07	0.06	1	aa
OE1-4	0.87	0.79	1	Aa

Note: The relative quantification and the absolute quantification was calculated according to the method described previously (Fan et al 2016). Both methods were used to calculate the copy numbers in over-expression plants. One copy insertion T0 plants (Aa) were harvested and grown to generate T1 plants. T1 plants of AA genotype were taken as being homozygous, T1 plants of aa genotype were taken as false positive (SDWT); T4 generation of OE1-1 (AA) renamed as pOsNAR2.1-OsNAR2.1 in WGBS experiment; T4 generation of OE1-3 (aa) renamed as SDWT in WGBS experiment. The phenotype of all transgenic lines and the identification in DNA, RNA and protein levels were described in Chen et al. 2017 Plant Biotech. J. For this experiment, OE1-1 renamed as pOsNAR2.1-OsNAR2.1 and OE1-3 line renamed as SDWT.

Commented [M1]: Please format tables using Microsoft Word.

Table can not be presented in the form of pictures, Please format it as editable three-line tables using Microsoft Word so that we can edit in the production process.

Commented [Xiaoru2R1]: Thank you, we changed all tables with Word

Table S2. Comparison of agronomic traits between WYJ7, SDWT and *pOsNAR2.1-OsNAR2.1* transgenic lines.

Genotype	WYJ7	CK	SDWT	*pOsNAR2.1-OsNAR2.1*
Plant height (cm)	83.81±1.44b	78.31±2.11c	83.12±1.06b	87.74±2.81a
Total tiller number per plant	20.48±1.48b	21.02±2.43b	21.14±1.95b	26.78±0.99a
Panicle length (cm)	13.78±0.77b	12.91±0.58b	13.24±0.62b	15.67±1.07a
Seed setting rate (%)	72.67±4.35b	63.12±2.24c	70.33±5.58bc	83.04±3.62a
Grain weight (g/panicle)	2.32±0.12b	1.98±0.06c	2.47±0.09b	3.01±0.07a
Grain number per panicle	130.67±11.20b	102.48±9.67c	127.56±9.86b	153.8±10.43a
Yield (g/plant)	21.56±2.12b	15.28±1.27c	27.18±1.34b	32.14±2.89a

Note: Statistical analysis of SDWT and *pOsNAR2.1-OsNAR2.1* lines were performed on data derived from the T4 generation. For each mean, error: SD (n = 5 plants). Significant differences between different lines are indicated by different letters (P < 0.05, one-way ANOVA).

Table S3. Comparison of agronomic traits between wild-type of NP, RNAi and *pUbi-OsNAR2.1* transgenic lines.

Genotype	NP	RNAi	*pUbi-OsNAR2.1*
Plant height (cm)	86.27±4.23b	70.61±5.94c	101.25±5.37a
Total tiller number per plant	24.62±1.76a	25.78±1.95a	23.10±1.81a
Panicle length (cm)	16.13±0.69a	14.76±0.57b	16.59±0.68a
Grain weight (g/panicle)	1.17±0.07b	0.88±0.06c	1.84±0.10a
Grain number per panicle	60.46±7.51b	44.12±4.90c	99.06±8.31a
Seed setting rate (%)	71.67±4.31b	48.67±4.07c	86.17±3.49a
Yield (g/plant)	21.56±2.12b	10.54±1.05c	29.10±2.71a

Note: Statistical analysis was performed on data derived from the T8 generation transgenic lines. error: SD (n = 5 plants). Significant differences between different lines are indicated by different letters (P < 0.05, one-way ANOVA).

Table S4. Character of methylation status and agronomic traits of CK.

	WYJ7				CK			
	Line1	Line2	Line3	AVE±SD	Line1	Line2	Line3	AVE±SD
mC	396,005.25	380,986.58	406,068.46	$(3.94\pm0.12)X10^5$a	340,144.13	3441,38.92	375,678.51	$(3.35\pm0.19)X10^6$b
mCG Ratio (%)	46.93	48.58	48.6	48.04 ± 0.96a	47.76	50.4	46.75	48.30 ± 1.88a
mCHG Ratio (%)	26.07	26.85	26.65	26.52 ± 0.41a	24.09	25	23.79	24.29 ± 0.63b
mCHH Ratio (%)	27.01	24.57	24.75	25.44 ± 1.36a	28.15	24.6	29.46	27.40 ± 2.51a
Plant height(cm)	83.43	82.34	84.30	83.36 ± 0.98a	77.27	80.00	79.82	79.03 ± 2.29b
Total tiller number per plant	20.00	19.00	21.00	20.00 ± 1.00a	19.00	22.00	24.00	21.67 ± 2.51a
Panicle length (cm)	13.35	14.25	13.54	13.71 ± 0.47a	12.14	13.35	12.55	12.68 ± 0.62b
Seed setting rate (%)	71.25	70.44	68.25	69.98 ± 1.55a	60.21	65.42	64.45	63.36 ± 2.77a
Grain weight (g/panicle)	2.55	2.45	2.14	2.38 ± 0.21a	1.89	2.05	2.10	2.01 ± 0.11a
Grain number per panicle	140.35	135.35	133.35	136.35 ± 3.61a	102.45	100.47	98.45	100.45 ± 2.00b
Yield (g/plant)	27.44	26.46	28.45	27.45 ± 0.99a	17.54	18.53	14.64	16.90 ± 2.02b

Note: AVE indicated average, SD indicated standard deviation. Different letters indicated significant differences between CK and WYJ7 (P<0.05, two-pair T-test).

Table S5. The TAIL PCR primers and reaction programs for the identification of the insertion location of *pOsNAR2.1-OsNAR2.1* and *pUbi-OsNAR2.1* lines.

TAIL PCR Primers	
SP1	GCATGACGTTATTTATGAGATGGG
SP2	AATATAGCGCGCAAACTAGG
SP3	GCGGTGTCATCTATGTTAC
AD1	TGWGNAGWANCASAGA
AD2	WAGTGNAGWANCANAGA
AD3	STTGNTASTNCTNTGC

AD4	WCAGNTGWTNGTNCTG

REACTION PROGRAM (Control Method: CALCULATED)

TAIL 1 °	TAIL 2 °	TAIL 3 °
1=4 °for 2 min.<br>2=92 °for 2 min.<br>3=95 °for 1 min.<br>4=94 °for 30 sec.<br>5=66 °for 1 min.<br>6=72 °for 2 min.<br>7=Go to step 4 for 5 more cycles<br>8=94 °for 30 sec.<br>9=30 °for 3 min.<br>10=Ramp for 72 ° at 0.2 °/sec, 72 °for 2 min. 30 sec.<br>11=94 °for 5 sec.<br>12=66 °for 1 min.<br>13=72 °for 2 min.<br>14=94 °for 5 sec.<br>15=66 °for 1 min.<br>16=72 °for 2 min.<br>17=94 °for 5 sec.<br>18=44 °for 1 min.<br>19=72 °for 2 min.<br>20=Go to step 12, for 15 more cycles<br>21=72 °for 5 min.<br>22=4 °forever<br>23=END	1=95 °for 2 min.<br>2=94 °for 10 sec.<br>3=66 °for 1 min<br>4=72 °for 2 min.<br>5=94 °for 10 sec.<br>6=66 °for 1 min.<br>7=72 °for 2 min.<br>8=94 °for 10 sec.<br>9=44 °for 1 min.<br>10=72 °for 2 min.<br>11=Go to step 2, for 12 more, for 12 more cycles<br>12=72 °for 5 min.<br>13=4 °forever<br>14=END	1=95 °for 2 min.<br>2=94 °for 10 sec.<br>3=60 °for 1 min.<br>4=72 °for 2 min.<br>5=Go to step 2, for 19 more cycles<br>6=72 °for 5 min.<br>7=4 °forever<br>8=END

Table S6 Primers used for RT-PCRs

Gene name	Type of PCR	Primer	Sequence
OsNAR2.1<br>AP004023	quantitative real-time PCR	5’（F）	GTCGTCGAGAAGCGCAAGA
		5’（R）	GTCCACTGAAGCTGCGAACTT
OsActin<br>AB047313	quantitative real-time PCR	5’（F）	AGGATTACCATGGCCTCAAGAG
		5’（R）	CGCGTATCTTCCCCATGAAG

Table S7. Total mC, valid coverage of methylated cytosine and calculated normalized mC.

Sample	Total mC	Valid coverage of methylated cytosine	Normalized mC	AVE
WYJ7-1	16,818,343	42.47	396005.25	
WYJ7-2	17,216,784	45.19	380986.58	394353.43a
WYJ7-3	17,123,907	42.17	406068.46	
CK-1	13,649,984	40.13	340144.13	
CK-2	14,278,324	41.49	344138.92	353320.52b
CK-3	14,320,865	38.12	375678.51	
SDWT-1	17,174,503	45.62	376468.71	
SDWT-2	16,948,669	43.12	393058.19	382312.66a
SDWT-3	17,874,189	47.36	377411.09	
pOsNAR2.1-OsNAR2.1-1	15,291,559	43.14	354463.58	
pOsNAR2.1-OsNAR2.1-2	13,969,545	42.56	328231.79	346512.99b
pOsNAR2.1-OsNAR2.1-3	16,300,615	45.68	356843.59	

Note: AVE indicated average of normalized mC in samples.
